# Allometry predicts trabecular bone structural properties in the carnivoran jaw joint

**DOI:** 10.1371/journal.pone.0202824

**Published:** 2018-08-24

**Authors:** M. Aleksander Wysocki, Z. Jack Tseng

**Affiliations:** Graduate Program in Computational Cell Biology, Anatomy, and Pathology, Department of Pathology and Anatomical Sciences, Jacobs School of Medicine and Biomedical Sciences, University at Buffalo, Buffalo, New York, United States of America; Drexel University, UNITED STATES

## Abstract

Because overall cranial morphology-biomechanics linkage in carnivorans is significantly influenced by both feeding and non-feeding ecological variables, whole-skull mechanical performance measures may be less sensitive to feeding ecology than regional characteristics within the skull. The temporomandibular joint could be one regional characteristic that is highly sensitive to feeding ecology considering that this joint is used in prey capture, food processing, and experiences compressive loading during mastication. Through 3D model construction, 3D printing, and compression tests, morphological and mechanical performance measures were determined for the temporomandibular joint trabecular bone structure of 40 species representative of the phylogenetic and ecology diversity of Carnivora. Remarkably, the results indicate that relative fill volume, relative structural complexity, elastic modulus, and relative maximum compressive strength of trabecular bone structure are not significantly related to phylogeny or ecology. The results reveal that morphological and mechanical performance attributes of trabecular bone structure are primarily influenced by body size, and that positive centroid size allometry and positive body mass allometry are present for structural complexity. The lack of feeding ecological signal in dorso-ventral compressive loading of temporomandibular joint models indicates that carnivoran temporomandibular joint trabecular structures may not undergo significant differential remodeling as an evolutionary response to different mechanically demanding feeding tasks.

## Introduction

Historically it has been assumed that cranial structure-biomechanics relationships are primarily influenced by feeding ecology in carnivorans, but recent results from geometric morphometrics and finite element analysis suggest that carnivoran cranial morphology is related to both feeding and non-feeding variables [1]. The carnivoran skull must meet myriad functional demands besides prey capture and feeding, which may suggest that more suitable attributes for evaluating feeding ecology are mechanical performance measures from specific anatomical structures (e.g., the temporomandibular joint, or TMJ) that are directly associated with prey capture and food processing.

The mandibular condyle of the temporomandibular joint in mammals is likely to be under selection involving masticatory mechanical loads, given that previous research utilizing rosette strain gages in *Sus* (domestic pigs) shows that the mandibular condyle is subject to loading during mastication and that these forces are mostly compressive [2]. In addition, the finding that *Oryctolagus cuniculus* (domestic rabbits) subjected to over-use diets display greater biomineralization levels in the mandibular condyle than are observed in under-use diet *O*. *cuniculus* individuals implies that the mandibular condyle bone composition is responsive to level of mechanical loading over individual lifespans [3]. Furthermore, diets that require lower mechanical loading appear to be associated with increased rates of temporomandibular joint disorders and tooth development pathologies in human populations suggesting that biomechanical feedback contributes to the maintenance of harmonized oral tissue development [4]. Upon consideration that the study of human mandibular condyles shows that trabecular bone experiences much greater ranges of stresses and strains than does the cortical bone [5], temporomandibular joint trabecular bone structure might offer a useful structure-performance measure for assessing feeding ecology.

Here, we use a 3D modeling, 3D printing, and material testing approach to test the following hypothesis regarding the structure-performance relationship in the carnivoran TMJ:

### H_0_

Mechanical demands of prey capture and food processing have a significant influence on the mechanical properties of temporomandibular joint trabecular bone structure across species in Carnivora. The compressive load experienced by the carnivoran temporomandibular joint should be associated with evolutionary adaptation to mechanical demands of feeding.

## Materials & methods

**Fig 1:**
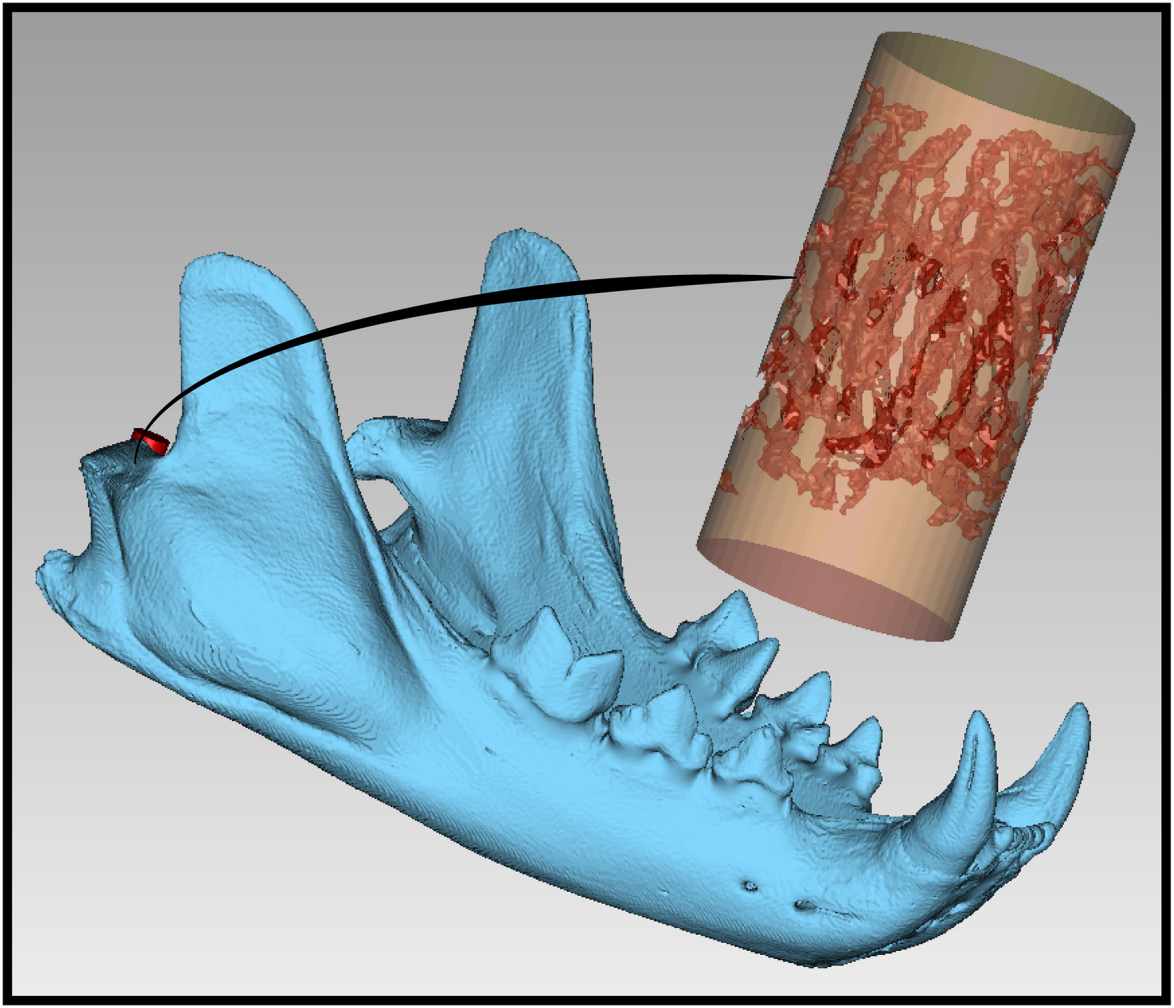
Standardization of carnivoran trabecular bone structure samples. 3D model of trabecular bone structure extracted from the mandibular condyle and standardized for mechanical property testing. 3D Models of Bobcat *Lynx rufus* (AMNH 24225) shown.

Trabecular bone structure from the central region of the mandibular condyle (i.e., a heterogeneous tissue that withstands compressive loading during mastication) was examined in 40 carnivoran species that are representative of the phylogenetic and ecological diversity of the Order (S1 Table) [2]. High resolution CT-scans were obtained with the GE v|tome|x s scanner at the American Museum of Natural History (AMNH). Most of the specimens scanned were adult, wild-caught individuals. The CT-image stacks were segmented using Dragonfly (Object Research Systems, Quebec, Canada) and 3D models were built in Geomagic Wrap (3D Systems, Rock Hill, South Carolina). Virtual “core samples” were taken from the mandibular condyle in the dorso-ventral orientation in the shape of a standardized cylinder. The height of a given cylinder was determined by using the maximum height of the articular surface from the central region of the mandibular condyle. Each cylinder diameter was set at 50% of its height to standardize the shape of the test sample. Using Boolean mesh operations in Geomagic Wrap, the 3-dimensional trabecular structure of mandibular condyle bone was cored using the cylindrical shape placed in the joint model. Each 3D model cylinder was then scaled to a height of 10mm and diameter of 5mm, thereby creating a standardized sample of carnivoran mandibular condyle trabecular bone structures to reduce error in the subsequent experimental data collection process (Fig 1).

Trabecular structure 3D models were imported into 3D Systems Sprint printing software. The specimen number was engraved on the superior surface of a given sample with a depth of 0.1mm. Samples were then printed using a 3D Systems ProJet MJP 2500 3D printer. All samples were printed using VisiJet M2R-CL material, which possesses a tensile strength of 35–45 MPa [6]. Although the rigid plastic print material does not replicate the material properties of actual mammalian bone, it is used as the standard model material in order to test the effect of trabecular bone structural differences on mechanical properties. Trabecular bone structural properties were measured by applying compressive load to each 3D-printed cylinder sample with a universal material tester. Five replicate prints of identical 3D models (n = 1 model for each species) were tested for each of the 40 species to account for uncertainty generated by variability of the 3D printing process. All sample tests began with a preload value of 10N to standardize the initial load onto the sample and were carried out with an average load rate of 0.6 mm/min using a manual crank and a watch. Data were measured using a Mark-10 ES-30 testing stand fitted with a force gauge (Model M3-500) and a Mitutoyo vertical caliper travel display. The force and vertical displacement data were recorded at a rate of 4 Hz using MesurGage.

Morphological data describing trabecular bone structure were measured using Geomagic Wrap. Relative fill volume (mm^3^) and relative structural complexity measured as relative surface area (mm^2^) were calculated using the 3D model that served as the basis for each 3D print. Raw data from compressive load tests were analyzed using Excel to obtain elastic modulus (MPa) and relative maximum compressive strength (N) for the trabecular bone structure of each species using the stress-strain curves of each sample test. Stress was calculated by dividing the recorded force by the surface area of the cylinder face (circle with radius of 2.5 mm, or area of 19.63 mm^2^) in contact with the compression plate of the testing machine. Strain was calculated by dividing the recorded vertical displacement distance (mm) by the original height of the cylinder sample (10 mm). Maximum compressive strength was then determined by identifying the maximum stress value. Elastic modulus was calculated by estimating the slope of the linear (elastic) region of the stress-strain curve. Because the test protocol involved standardizing the size of the test samples, centroid size information and body mass information were reintroduced into the test data by multiplying relative fill volume, relative structural complexity, and relative maximum compressive strength each by centroid size [1] or body mass [7] (e.g., Centroid Size x Relative Fill Volume = Centroid Size Included Volume).

Phylogenetic generalized least squares (PGLS) analyses were used to assess potential relationships of morphological and mechanical performance data with activity cycle, terrestriality, habitat breadth, dietary breadth, trophic level, maximum longevity, age of sexual maturity, mean monthly precipitation, temperature, dietary mechanical demand, and suborder. The ecological data were obtained from the PanTHERIA database of mammalian ecological characteristics [7]. Additionally, the degree of allometry was determined using linear regressions of the natural logarithms of trabecular bone morphological and mechanical performance variables against body size measures (mandible centroid size and body mass). Expected isometry slope values were determined for the properties being assessed. A value of m = 1 was expected for trabecular bone volume compared to body size because both attributes increase in 3 dimensions, whereas m = ^2^/_3_ was expected for the surface area (structural complexity) because surface area increases within 2 dimensions while body size increases in 3 dimensions. It was expected that m = 1 for maximum compressive strength because the capacity for withstanding compressive force should increase uniformly with increase to body size given that the amount of material available to withstand loading would increase. Positive/negative allometry was defined as expected slope values failing to fall within the confidence intervals of the estimated slope coefficient from PGLS regression analysis.

**Fig 2:**
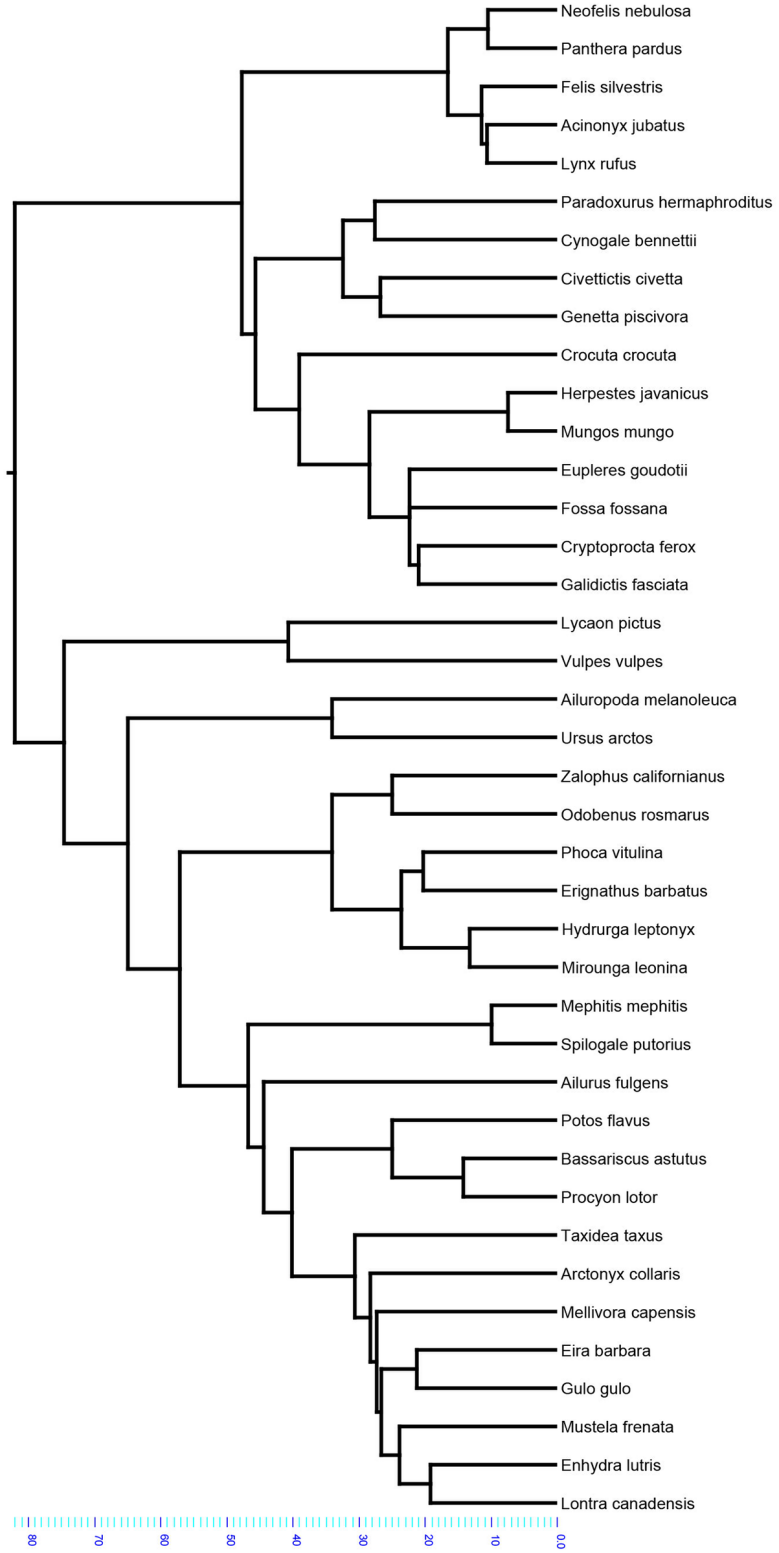
Consensus tree (principal analyses). Scale is in millions of years.

**Fig 3:**
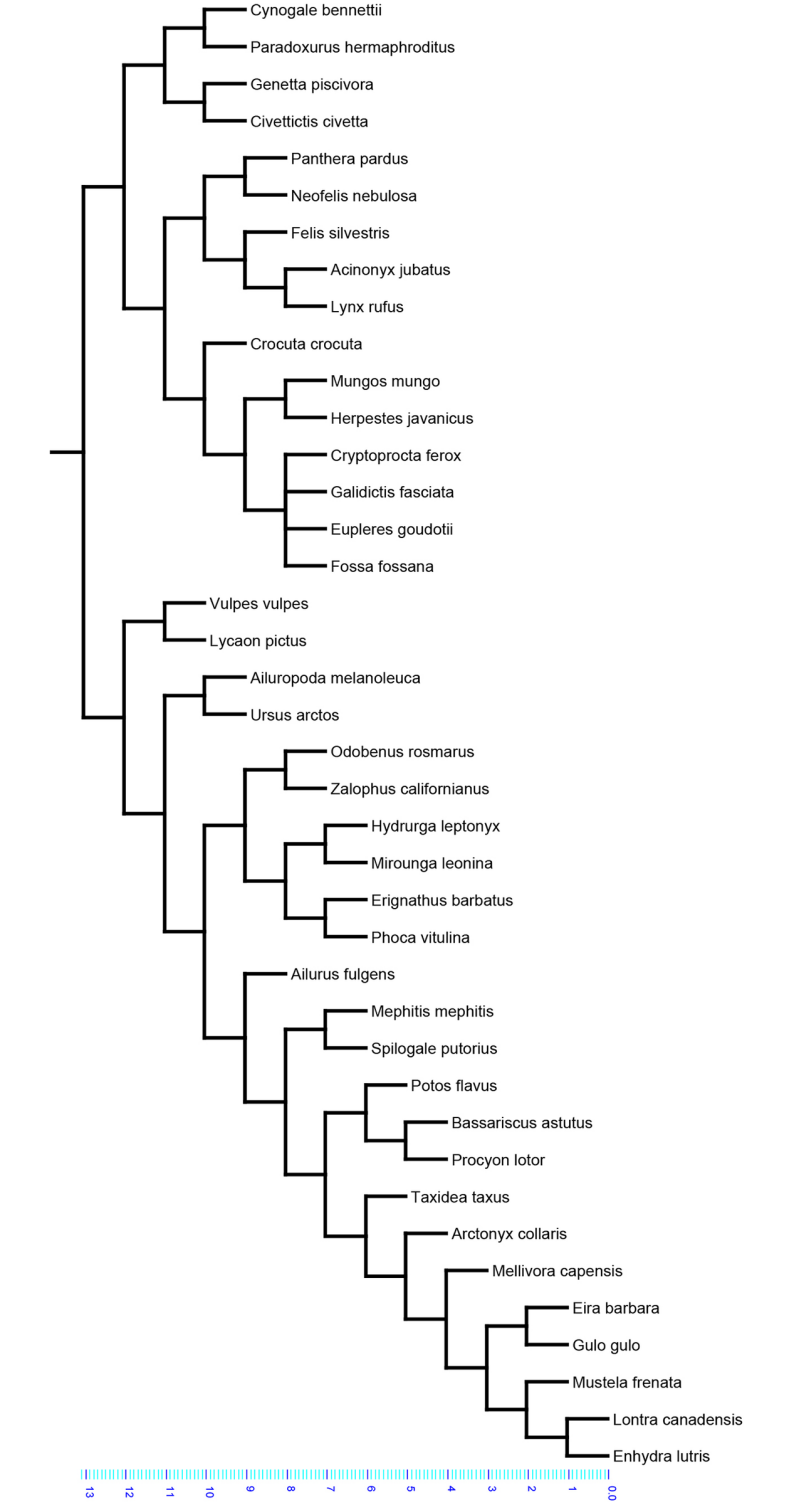
Uniform branch length tree (supplemental analyses). Scale is in arbitrary branch length units of one.

Data analysis was carried out using R in the phytools, nlme, ape, Geiger, Morpho, picante, geomorph, PHYLOGR, OUwie, and caper packages [8–18]. The phylogenetic topologies and branch lengths were constructed in Mesquite [19]. Principal analyses were run using a consensus tree (Fig 2) with branch lengths from 10kTrees Project (based on DNA sequence data) and supplemented with analyses from a uniform branch length configuration (Fig 3) adapted from [1].

## Results

### Morphological attributes of trabecular bone structure

The phylogeny-associated results of analyses using the molecular branch length configuration described below are consistent with the results of the analyses using the uniform branch length configuration (S2 and S3 Tables). *Mellivora capensis* (honey badger) displays the greatest relative fill volume for a trabecular bone structural sample, whereas *Bassariscus astutus* (ringtail) has the least relative fill volume (Table 1). The results indicate that centroid size included volume is positively correlated with maximum longevity and sexual maturity age (Table 2). Centroid size included volume also has a negative correlation with dietary mechanical demand. Centroid size corrected volume, however, is not correlated with any ecological variables. Similarly, body mass included volume is positively correlated with maximum longevity and sexual maturity age, as well as negatively correlated with dietary mechanical demand. Body mass corrected volume is not correlated with any variables.

**Table 1:** Morphological and mechanical performance data of trabecular bone structure.

Species	Specimen	Volume (mm^3^)	Structural Complexity (mm^2^)	Elastic Modulus (MPa)	Maximum Compressive Strength (N)
*Acinonyx jubatus*	AMNH185436	161.75	398.83	320.44	784.40
*Ailuropoda melanoleuca*	AMNH89079	108.33	831.48	88.28	95.20
*Ailurus fulgens*	AMNH145071	161.75	407.57	277.74	394.80
*Arctonyx collaris*	AMNH57373	144.51	522.66	185.62	151.20
*Bassariscus astutus*	AMNH135964	106.11	372.58	119.87	80.40
*Civettictis civetta*	AMNH51818	149.81	511.89	203.33	214.00
*Crocuta crocuta*	AMNH187777	132.86	642.28	202.07	270.80
*Cryptoprocta ferox*	AMNH100463	133.65	620.35	108.31	96.40
*Cynogale bennettii*	AMNH173509	143.43	474.15	247.48	225.20
*Eira barbara*	AMNH32065	153.55	441.41	238.75	224.40
*Enhydra lutris*	AMNH24186	163.92	456.06	263.99	483.60
*Erignathus barbatus*	AMNH98	157.53	486.86	219.20	190.80
*Eupleres goudotii*	AMNH100484	125.41	372.58	133.57	104.80
*Felis silvestris*	AMNH81233	123.73	503.54	150.70	108.00
*Fossa fossana*	AMNH188210	179.72	269.42	335.58	714.40
*Galidictis fasciata*	AMNH100479	125.69	337.99	111.13	85.20
*Genetta piscivora*	AMNH51514	144.46	367.27	298.13	376.00
*Gulo gulo*	AMNH182936	179.85	309.42	344.09	940.80
*Herpestes javanicus*	AMNH101655	159.90	513.76	292.79	488.00
*Hydrurga leptonyx*	AMNH34920	124.78	952.08	117.24	164.40
*Lontra canadensis*	AMNH254476	176.93	375.71	311.53	641.20
*Lycaon pictus*	AMNHVP24218	114.62	742.53	117.83	108.40
*Lynx rufus*	AMNH24225	162.04	502.80	294.03	479.60
*Mellivora capensis*	AMNH89011	180.89	383.51	327.67	710.80
*Mephitis mephitis*	AMNH172133	123.49	437.59	119.78	86.40
*Mirounga leonina*	AMNH48161	152.12	701.74	249.75	618.80
*Mungos mungo*	AMNH185177	135.90	379.55	201.40	135.60
*Mustela frenata*	AMNH60508	138.70	283.45	195.02	209.20
*Neofelis nebulosa*	AMNH22919	161.13	516.18	266.98	381.60
*Odobenus rosmarus*	AMNH19270	140.34	714.23	49.91	32.00
*Panthera pardus*	AMNH113745	151.85	677.72	231.71	96.40
*Paradoxurus hermaphroditus*	AMNH163602	141.70	531.49	171.80	176.40
*Phoca vitulina*	AMNH100	138.22	608.71	145.38	181.20
*Potos flavus*	AMNH239990	173.53	319.41	328.67	693.20
*Procyon lotor*	AMNH24815	134.27	357.53	188.73	133.20
*Spilogale putorius*	AMNH35207	167.16	306.86	306.67	466.40
*Taxidea taxus*	AMNH120577	152.79	539.36	231.21	258.80
*Ursus arctos*	AMNH34408	172.16	519.90	276.61	533.20
*Vulpes vulpes*	AMNH88713	126.39	565.86	126.65	105.60
*Zalophus californianus*	AMNH63946	167.08	408.00	292.19	453.20

**Table 2:** PGLS analyses of volume and ecological variables.

	Centroid Size Included Volume		Centroid Size Corrected Volume		Body Mass Included Volume		Body Mass Corrected Volume	
Variable	P	RC	P	RC	P	RC	P	RC
Activity Cycle	0.42	0.10	0.88	-0.01	0.29	0.45	0.93	0.00
Terrestriality	0.62	-0.08	0.24	0.07	0.50	-0.37	0.24	0.07
Habitat Breadth	0.95	0.01	0.49	0.03	0.88	0.07	0.56	0.03
Dietary Breadth	0.31	0.03	0.63	0.00	0.92	0.01	0.97	0.00
Trophic Level	0.37	-0.12	0.86	-0.01	0.93	0.04	0.76	0.01
Maximum Longevity	**0.00**	0.00	0.68	0.00	**0.00**	0.01	0.57	0.00
Sexual Maturity Age	**0.00**	0.00	0.76	0.00	**0.00**	0.00	0.51	0.00
Mean Monthly Precipitation	0.48	0.00	0.77	0.00	0.16	-0.01	0.88	0.00
Temperature	0.40	-0.08	0.47	0.00	0.49	0.00	0.54	0.00
Mechanical Demand	**0.00**	-0.45	0.51	-0.03	**0.00**	-1.52	0.37	-0.04
Suborder	0.42	-0.26	0.75	-0.02	0.43	-1.15	0.77	0.01

P = P-value, RC = Regression Coefficient. Statistically significant (alpha = 0.05 level) regressions shown in bold font.

Greatest relative structural complexity (952.0753 mm^2^) for mandibular condyle trabecular bone occurs in *Hydrurga leptonyx* (leopard seal) and the least relative structural complexity (269.4243 mm^2^) is apparent in *Fossa fossana* (Malagasy civet). The results indicate that centroid size included structural complexity is positively correlated with maximum longevity and sexual maturity age, as well as negatively correlated with dietary mechanical demand (Table 3). Comparable to centroid size included structural complexity, body mass included structural complexity exhibits positive correlations with maximum longevity and sexual maturity age, and a negative correlation with dietary mechanical demand. However, neither centroid size corrected structural complexity nor body mass corrected structural complexity have significant correlations with any of the ecological variables.

**Table 3:** PGLS analyses of structural complexity and ecological variables.

	Centroid Size Included Structural Complexity		Centroid Size Corrected Structural Complexity		Body Mass Included Structural Complexity		Body Mass Corrected Structural Complexity	
Variable	P	RC	P	RC	P	RC	P	RC
Activity Cycle	0.43	0.13	0.48	0.04	0.29	0.47	0.73	0.02
Terrestriality	0.19	-0.28	0.27	-0.09	0.33	-0.56	0.11	-0.14
Habitat Breadth	0.88	-0.02	0.80	-0.02	0.93	0.04	0.58	-0.04
Dietary Breadth	0.33	0.04	0.71	-0.01	0.87	0.02	0.62	-0.01
Trophic Level	0.30	-0.19	0.85	0.01	0.97	-0.02	0.94	0.00
Maximum Longevity	**0.00**	0.00	0.37	0.00	**0.00**	0.01	0.61	0.00
Sexual Maturity Age	**0.00**	0.00	0.60	0.00	**0.00**	0.00	0.74	0.00
Mean Monthly Precipitation	0.33	0.00	0.66	0.00	0.15	-0.01	0.84	0.00
Temperature	0.77	0.00	0.16	0.00	0.67	0.00	0.21	0.00
Mechanical Demand	**0.02**	-0.50	0.69	0.03	**0.00**	-1.55	0.93	-0.01
Suborder	0.46	-0.41	0.24	0.08	0.43	-1.31	0.31	0.08

P = P-value, RC = Regression Coefficient. Statistically significant (P = 0.05 level) regressions shown in bold font.

### Mechanical performance attributes of trabecular bone structure

Interestingly, the species with the maximum and minimum values for the morphological measurements (relative fill volume and relative structural complexity) are not the species that exhibit the maximum and minimum mechanical performance values. The greatest elastic modulus and greatest relative maximum compressive strength are displayed by *Gulo gulo* (wolverine); a species that wields durophagous dentition and is known to crush the bones of much larger species [7, 20]. From a purely structural perspective, *Odobenus rosmarus* (walrus) trabecular bone morphology appears to be very poor for withstanding compressive loading given that this species exhibits the minimum values for elastic modulus and relative maximum compressive strength. This finding may be related to the diet of *O*. *rosmarus* that consists primarily of soft invertebrates and to the great body size of this particular species [7, 21]. Incorporating mandible centroid size into the calculations yields different species for both the least and greatest maximum compressive strength values, which are *Galidictis fasciata* (broad-striped mongoose) and *Ursus arctos* (brown bear), respectively.

Potential relationships of structural mechanical performance data were explored using phylogenetic generalized least squares analyses with activity cycle, terrestriality, habitat breadth, dietary breadth, trophic level, maximum longevity, age of sexual maturity, mean monthly precipitation, temperature, dietary mechanical demand, suborder, relative structural complexity, and relative fill volume. As elastic modulus is by definition size independent, size inclusion and size correction are not required for elastic modulus analyses [22]. The results show that elastic modulus is positively correlated with relative fill volume and negatively correlated with relative structural complexity (Table 4). Centroid size included maximum compressive strength is positively correlated with relative fill volume and maximum longevity, as well as negatively correlated with dietary mechanical demand (Table 5). Centroid size corrected maximum compressive strength shows a positive correlation with relative fill volume and a negative correlation with relative structural complexity.

**Table 4:** PGLS analyses of elastic modulus and ecological variables.

Variable	P	RC
Activity Cycle	0.91	-2.39
Terrestriality	0.49	22.32
Habitat Breadth	0.99	0.45
Dietary Breadth	0.82	-1.27
Trophic Level	0.83	4.79
Maximum Longevity	0.59	-0.06
Sexual Maturity Age	0.14	-0.03
Mean Monthly Precipitation	1.00	0.00
Temperature	0.64	-0.07
Mechanical Demand	0.40	-23.28
Suborder	0.72	9.66
Surface Area	**0.00**	-0.28
Volume	**0.00**	3.73

P = P-value, RC = Regression Coefficient. Statistically significant (alpha = 0.05 level) regressions shown in bold font.

**Table 5:** PGLS analyses of maximum compressive strength and ecological variables.

	Centroid Size Included Maximum Compressive Strength		Centroid Size Corrected Maximum Compressive Strength		Body Mass Included Maximum Compressive Strength		Body Mass Corrected Maximum Compressive Strength	
Variable	P	RC	P	RC	P	RC	P	RC
Activity Cycle	0.68	0.10	0.96	-0.01	0.31	0.52	0.73	0.08
Terrestriality	0.86	-0.07	0.59	0.18	0.71	-0.25	0.65	0.15
Habitat Breadth	0.92	0.03	0.91	0.03	0.70	0.21	0.92	0.02
Dietary Breadth	0.93	-0.01	0.98	0.00	0.77	0.04	0.65	-0.03
Trophic Level	0.74	-0.09	0.90	-0.03	0.93	-0.05	0.70	0.09
Maximum Longevity	**0.03**	0.00	0.99	0.00	**0.00**	0.01	0.82	0.00
Sexual Maturity Age	0.17	0.00	0.34	0.00	**0.00**	0.00	0.31	0.00
Mean Monthly Precipitation	0.50	0.00	0.84	0.00	0.25	-0.01	0.63	0.00
Temperature	0.28	0.00	0.39	0.00	0.35	0.00	0.37	0.00
Mechanical Demand	**0.02**	-0.73	0.27	-0.30	**0.00**	-1.86	0.16	-0.40
Suborder	0.37	-0.29	0.79	-0.07	0.45	-1.03	1.00	0.00
Surface Area	0.91	0.00	**0.01**	0.00	0.24	0.00	**0.02**	0.00
Volume	**0.00**	0.04	**0.00**	0.03	**0.00**	0.05	**0.00**	0.03

P = P-value, RC = Regression Coefficient. Statistically significant (alpha = 0.05 level) regressions shown in bold font.

Body mass included maximum compressive strength is positively correlated with maximum longevity, age of sexual maturity, and relative fill volume. Also, body mass included compressive strength is negatively correlated with mechanical demand. Similar to centroid size corrected maximum compressive strength, the only significant relationships for body mass corrected maximum compressive strength are a positive correlation with relative fill volume and a negative correlation with relative structural complexity. Although maximum compressive strength shows correlations with some ecological variables, the analyses that account for body size via centroid size or body mass show that maximum compressive strength only has significant relationships with relative fill volume and relative structural complexity. Overall, these results indicate that increases to elastic modulus and maximum compressive strength are associated with increases to trabecular bone relative fill volume. Conversely, elastic modulus and maximum compressive strength decrease as the relative structural complexity of trabecular bone increases.

### Quantification of allometry

The degree to which allometry influences trabecular bone of the mandibular condyle is further evaluated through linear regressions of the natural logarithms (ln) of morphological and mechanical performance characteristics versus ln centroid size and ln body mass (Table 6). The results show that elastic modulus and centroid size, as well as elastic modulus and body mass, do not have a significant relationship. These outcomes are consistent with expectations given that by definition elastic modulus is independent of size [22]. Relative fill volume correlates with centroid size and with body mass, as expected. The relationships between volume and body size measures do not differ significantly from isometry. Similarly, maximum compressive strength exhibits correlations with centroid size and body mass, and the degrees of allometry do not significantly differ from isometry. Trabecular bone structural complexity also has correlations with centroid size and with body mass. The results indicate that positive allometry exists for structural complexity even at a 99% confidence interval for the analysis using centroid size and for the analysis based on body mass.

**Table 6:** PGLS analyses of trabecular bone structure attributes versus body size.

		P	RC	SE	CI Lower Limit	CI Upper Limit	Expected RC
Volume	Centroid Size	**0.00**	1.00	0.05	0.88	1.12	1.00
Structural Complexity	Centroid Size	**0.00**	1.42	0.07	1.25	1.60	0.67*
Elastic Modulus	Centroid Size	0.30	-0.15	0.15	-0.53	0.22	0.00
Maximum Compressive Strength	Centroid Size	**0.00**	0.94	0.27	0.26	1.63	1.00
Volume	Body Mass	**0.00**	1.01	0.01	0.98	1.03	1.00
Structural Complexity	Body Mass	**0.00**	1.10	0.02	1.05	1.14	0.67*
Elastic Modulus	Body Mass	0.38	-0.03	0.04	-0.12	0.06	0.00
Maximum Compressive Strength	Body Mass	**0.00**	1.01	0.07	0.83	1.18	1.00

P = P-value, RC = Regression Coefficient, SE = Standard Error, CI = Confidence Interval. Statistically significant (alpha = 0.05 level) regressions shown in bold font.

*Statistically significant positive/negative allometry (expected value of isometry fails to fall within 99% confidence interval of the actual value).

## Discussion

The results pertaining to the relationships of morphological and mechanical performance attributes are essentially consistent with expectations. Elastic modulus and relative maximum compressive strength have positive correlations with relative fill volume, which matches expectations because greater amounts of material through which force can be distributed within a standard set of dimensions should be associated with greater stiffness and strength. The negative correlations between relative structural complexity and elastic modulus, as well as between relative structural complexity and relative maximum compressive strength, are also consistent with expectations for how these trabecular bone structures should respond to compressive loading. When adding compressive loads to rigid structures, a highly complex structure made up of numerous smaller load bearing supports would be expected to fail overall shortly after the weakest support undergoes fracture. In contrast, a second structure made up of exactly the same amount of material, but in the form of a completely solid column, should be capable of withstanding greater compressive loading because the overall structure is not dependent on the structural integrity of a smaller component. All in all, the mechanical performance results for trabecular bone structures are consistent with expectations for how those structures function under compressive loading.

**Fig 4:**
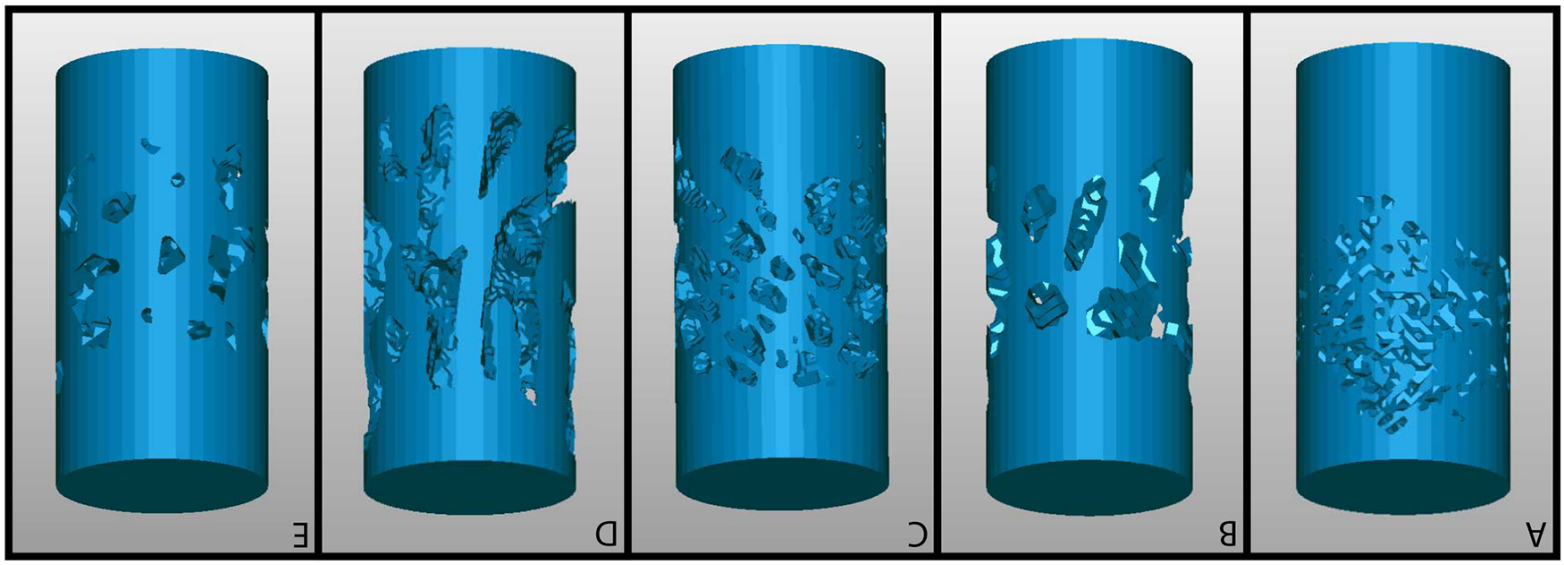
Scaled 3D models of carnivoran trabecular bone structure. (A) Brown bear *Ursus arctos*. (B) Raccoon *Procyon lotor*. (C) California sea lion *Zalophus californianus*. (D) Aquatic genet *Genetta piscivora*. (E) Kinkajou *Potos flavus*.

Although many different structures were evident in carnivoran trabecular bone (Fig 4), neither morphological measures nor mechanical performance measures show significant correlations with suborder or with any ecological variables after correcting for body size. Therefore, the results call for the rejection of the hypothesis that mechanical demands of prey capture and food processing have a significant effect on the mechanical properties of temporomandibular joint trabecular bone structure (H_0_). These findings are consistent with results from a study on three platyrrhine primate taxa that suggest mandibular condyle trabecular bone morphological characteristics do not significantly vary despite dissimilar dietary behavior [23]. Nevertheless, the discovery that temporomandibular joint trabecular bone structure is not significantly associated with feeding ecology is very striking given the observable involvement of the temporomandibular joint in prey capture and food processing, experimental results showing that the mandibular condyle experiences substantial compressive loading during mastication, and evidence that dietary mechanical demands influence mandibular condyle biomineralization levels as well as synchronized development of oral tissues [2–5].

These unanticipated results might suggest that ecological variables do not require drastically different temporomandibular joint mechanical capabilities in carnivorans. An alternative explanation is the possibility that selective pressures on trabecular bone structure from feeding ecology may actually be diminished by the mammalian mandibular condyle bone remodeling plasticity within an individual’s lifespan [3]. That is, the capacity of temporomandibular joint bone to change in response to mechanical demands experienced by the individual may be so responsive that trabecular bone structure is not primarily influenced by the mechanical demands of feeding ecology. It is also plausible that significant relationships exist between the temporomandibular joint and ecological variables, but the signals could be masked by this particular methodological approach. The current study specifically evaluates the mechanical performance of structure by testing all of the trabecular bone structural samples in the same rigid plastic material. This material (VisiJet M2R-CL) has a tensile strength of 35 to 45 MPa, whereas *Ursus americanus* tibia cortical bone has a tensile strength of 166 to 198 MPa [6, 24]. Thus, an inherent limitation is that the mechanical performance data cannot account for variations in the anisotropic material properties of bone between species; namely, that bone is a composite material consisting of a rigid mineralized component and a pliable collagen component [25]. Future research should evaluate the influence of mineralization levels and collagen levels on the mechanical performance of similar trabecular bone structures to further test whether or not trabecular bone of the temporomandibular joint is related to ecological variables.

The results indicate that centroid size allometry and body mass allometry are significantly correlated with temporomandibular joint trabecular bone structural properties. The positive allometry for structural complexity, which is a morphological attribute that is associated with both lower elastic modulus and lower maximum compressive strength, seems to suggest that carnivoran species of larger body size possess mandibular condyle trabecular bone morphology that is not structurally optimized for withstanding high compressive loads. This observation could reflect the relatively reduced mechanical demands in larger species (e.g., eating prey and/or experiencing masticatory forces that are relatively smaller compared to their size) and/or osteological growth mechanisms that maintain comparable trabecular bone subcomponent structures regardless of species body size. Body size and other non-feeding variables have been shown to influence the overall cranial morphology of Carnivora [1] and the results of this investigation demonstrate that even specific performance measures from the carnivoran temporomandibular joint are principally determined by non-feeding variables (i.e., size).

Lastly, the findings of this study are valid in a cross-species comparative context given the focus on broad taxonomic sampling (across 14 families) rather than multiple individuals of the same species (n = 1 individual per species). Bone functional adaptation theory indicates that the trabecular arrangement reflects stereotypical loading regimes experienced by the individual [26]. Therefore, some degree of intraspecific variation according to behavioral, geographic, or ontogenetic differences in jaw loading regimen are expected to be present. Future studies should investigate the extent to which intraspecific variations characterize ecological and developmental differences between individuals of the same species. It is possible that allometry may be the main predictor of TMJ trabecular bone structural properties in Carnivora as a whole, whereas subtle growth or ecological signals may be captured at the intraspecific level.

## Conclusions

Upon consideration that the temporomandibular joint is involved in prey capture and food processing, that the mandibular condyle experiences compressive loading during mastication, and that biomechanical feedback appears to influence mandibular condyle biomineralization levels and coordination of oral tissue development, the morphology and mechanical performance of this structure appeared to have great potential to provide engineering-based measures of feeding ecology [2–5]. The results of the current investigation show that temporomandibular joint trabecular bone structure morphological and mechanical performance measures are not significantly associated with feeding ecology variables across Carnivora. It appears that size allometry is the principal factor that influences temporomandibular joint trabecular bone structure’s relative fill volume, relative structural complexity, elastic modulus, and relative maximum compressive strength. Notably, positive allometry occurs for structural complexity of temporomandibular joint trabecular bone. Future research should explore temporomandibular joint trabecular bone structural properties in additional mammalian taxa to see if these patterns hold across mammals in general, and in ontogenetic and geographical intraspecific samples to see if patterns differ at the individual level.

## Supporting information

S1 Table Ecological traits compiled from PanTHERIA database. (CSV)

S2 Table Regressions from uniform branch length configuration analyses. (XLSX)

S3 Table Allometry analysis results from uniform branch length configuration analyses. (XLSX)
